# Supplementary material for: Maternal liver-related symptoms during pregnancy in primary sclerosing cholangitis
Source: JHEP Rep. 2023 Oct 31;6(1):100951. doi: 10.1016/j.jhepr.2023.100951 (PMC10711472; doi:10.1016/j.jhepr.2023.100951)
Supplement: Multimedia component 1 [file mmc1.pdf]

Maternal liver-related symptoms during pregnancy in primary sclerosing cholangitis

Jeremy S Nayagam, Tobias J Weismüller, Piotr Milkiewicz, Karolina M Wronka, Emil Bik, Christoph Schramm, Katja Fuessel, Taotao Zhou, Johannes Chang, Martti Färkkilä, Ylva Carlsson, Anastasia Lundman, Nora Cazzagon, Giorgia Corrà, Eirini Rigopoulou, George N Dalekos, Aiva Lundberg Båve, Annika Bergquist, Karim Ben Belkacem, Marco Marzioni, Martina Mancinelli, Xavier Verhelst, Hanns-Ulrich Marschall, Michael A Heneghan, Deepak Joshi, the International PSC Study Group (IPSCSG)

Table of contents

Fig. S1……………………………………………………..…………………………..…2

Fig. S2…………………………………..…………………………………………………3

Table S1……………………………………………………………………………………5

Table S2……………………………………………………………………………………6

Table S3……………………………………………………………………………………7

Table S4……………………………………………………………………………………9

Table S5…………………………………………………………………………..…....10

Fig. S1. Study design and subject disposition.

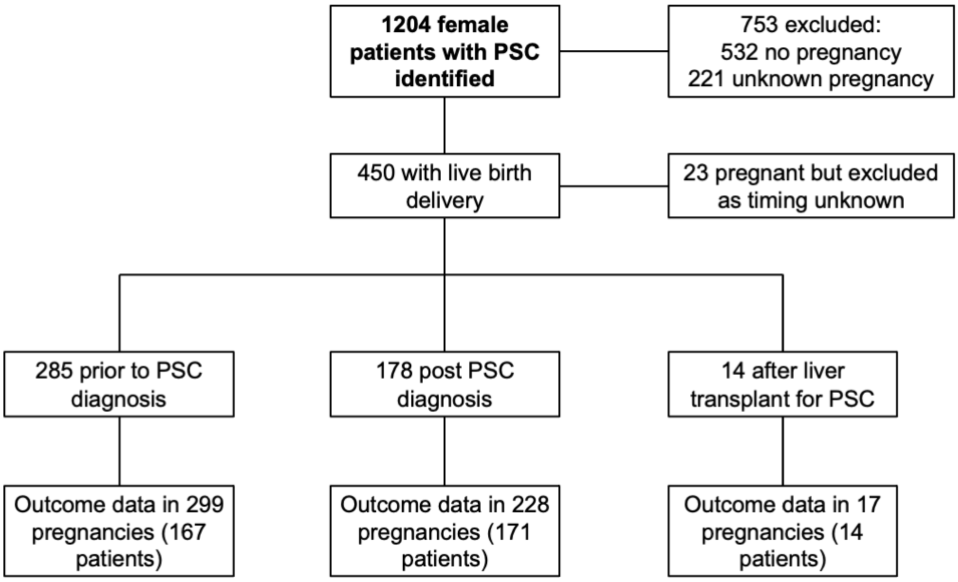

Fig. S2A. Cumulative incidence of clinical events with Kaplan-Meier estimates for time to liver transplantation or death (transplant-free survival) from time of pregnancy prior to PSC diagnosis. 20 year transplant-free survival in those with liver abnormalities in pregnancy (log-rank, P=0.01).

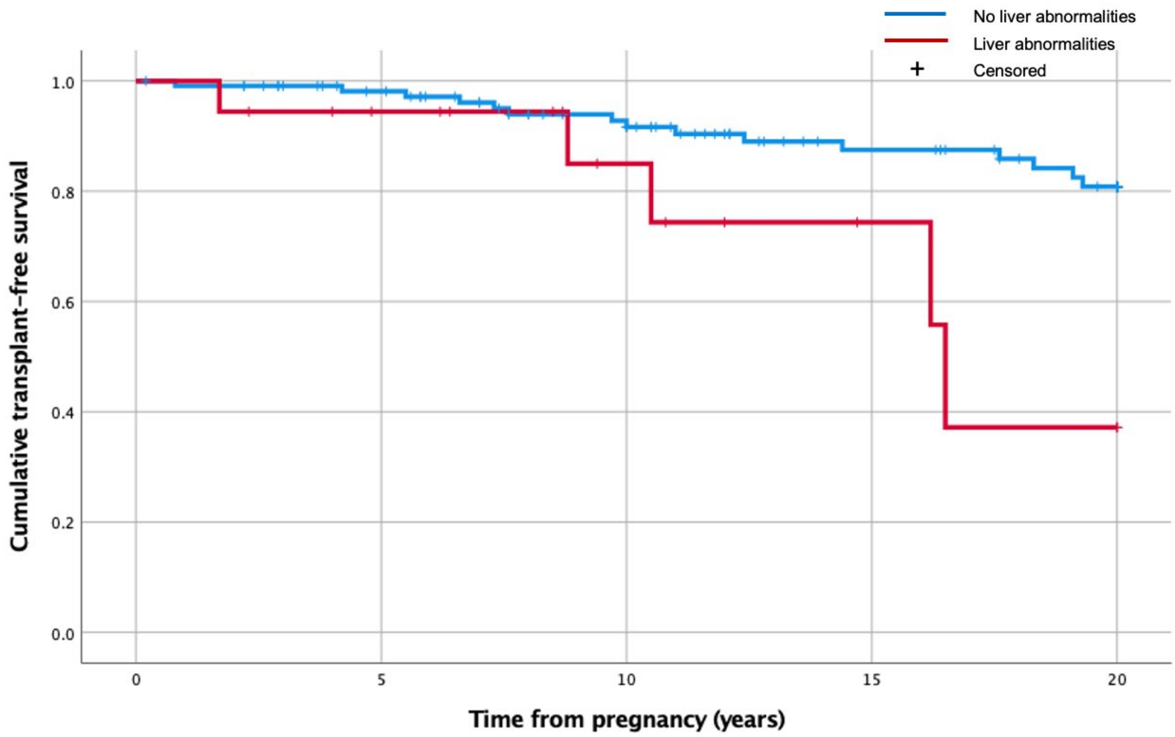

At risk					
Liver abnormalities	*18*	14	8	4	2
No liver abnormalities	*113*	100	81	58	47

Fig. S2B. Cumulative incidence of clinical events with Kaplan-Meier estimates for time to liver transplantation or death (transplant-free survival) from time of PSC diagnosis in those with pregnancy prior to diagnosis. 20 year transplant-free survival in those with liver abnormalities in pregnancy (log-rank, P=0.21).

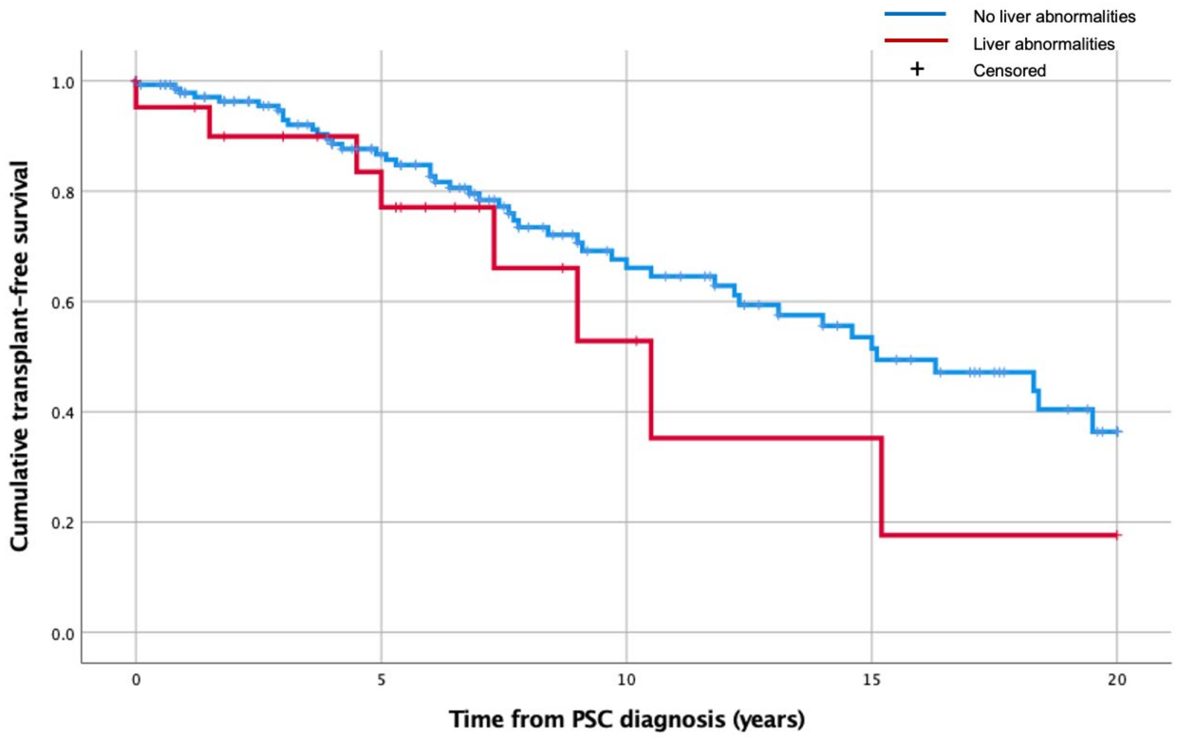

At risk					
Liver abnormalities	21	13	4	2	1
No liver abnormalities	*146*	90	44	26	7

Table S1. Clinical characteristics of subgroup of patients who were pregnant prior to their diagnosis of PSC (n=167).

	N=167
**Age at delivery, median (IQR), years**	28.7 (25.2-31.8),
**Age at PSC diagnosis, median (IQR), years**	38.8 (32.4-48.1)
**Interval between delivery and PSC diagnosis, median (IQR), years**	5.8 (1.7-17.1)
**Large duct**	132 (79.0%)
**Small duct**	14 (8.4%)
**PSC/AIH overlap**	19 (11.4%)
**IgG4-SC**	2 (1.2%)
**IBD**	85 (50.9%)

Abbreviations: PSC, primary sclerosing cholangitis; AIH, autoimmune hepatitis; IgG4-SC, immunoglobulin G4 sclerosing cholangitis; IBD, inflammatory bowel disease.

Continuous variables are expressed as median (IQR), categorical variables are represented as number (%).

Table S2. Clinical characteristics of subgroup of patients who were pregnant after their diagnosis of PSC (n=178).

	N=178
**Age at PSC diagnosis, median (IQR), years**	26.8 (21.5-30.7)
**Age at delivery, median (IQR), years**	32.3 (28.7-35.7)
**PSC duration at time of delivery, median (IQR), years**	4.4 (2.2-8.9)
**Large duct**	146 (82.0%)
**Small duct**	8 (4.5%)
**PSC/AIH overlap**	24 (13.5%)
**IBD**	122 (66.5%)

Abbreviations: PSC, primary sclerosing cholangitis; AIH, autoimmune hepatitis; IBD, inflammatory bowel disease.

Continuous variables are expressed as median (IQR), categorical variables are represented as number (%).

Table S3. Univariate analysis of continued UDCA use during pregnancy and change in liver blood tests from baseline values.

	UDCA not used previously or stopped	UDCA continued	P
**Bilirubin**			
Normal prior to conception	33/38 (86.8%)	53/65 (81.5%)	0.48
>25% rise from baseline during pregnancy	4/34 (11.8%)	10/51 (19.6%)	0.34
>25% rise post from baseline after delivery	8/32 (25.0%)	17/53 (32.1%)	0.49
**ALP**			
Normal prior to conception	19/42 (45.2%)	33/68 (48.5%)	0.74
>25% rise from baseline during pregnancy	7/36 (19.4%)	16/55 (29.1%)	0.30
>25% rise post from baseline after delivery	17/36 (47.2%)	27/58 (46.6%)	0.95
**ALT**			
Normal prior to conception	9/22 (40.9%)	24/46 (52.2%)	0.39
>25% rise from baseline during pregnancy	2/20 (10.0%)	7/41 (17.1%)	0.70
>25% rise post from baseline after delivery	8/29 (27.6%)	12/34 (35.3%)	0.51
**AST**			
Normal prior to conception	14/32 (43.8%)	12/42 (28.6%)	0.18
>25% rise from baseline during pregnancy	5/30 (16.7%)	6/32 (18.8%)	0.83

>25% rise post from baseline after delivery	10/18 (55.6%)	16/39 (41.0%)	0.31
**GGT**			
Normal prior to conception	9/27 (33.3%)	16/51 (31.4%)	0.86
>25% rise from baseline during pregnancy	**7/23 (30.4%)**	**3/35 (8.6%)**	**0.04**
>25% rise post from baseline after delivery	9/24 (37.5%)	8/39 (20.5%)	0.14

Abbreviations; UDCA, ursodeoxycholic acid; ALP, alkaline phosphatase; ALT, alanine aminotransferase; AST, aspartate aminotransferase; GGT, gamma-glutamyl transferase.

Categorical variables are represented as number (%), or where data is missing number/number available (%). Subgroup differences were analysed by Chi-squared or Fisher’s exact tests.

Table S4. Reported symptoms during pregnancy after PSC diagnosis (n=228).

	N=228
**Clinical diagnosis of intrahepatic cholestasis of pregnancy**	33 (14.5%)
**Pruritus**	
Stable or improved from baseline	33 (14.5%)
Worsened from baseline	23 (10.1%)
Newly developed	34 (14.9%)
**Cholangitis**	
Treated with antibiotics as outpatient	3 (1.3%)
Treated with antibiotics as inpatient	1 (0.4%)
**Any worsening liver-related symptom during pregnancy**	66 (28.9%)

Categorical variables are represented as number (%).

Table S5. Immunosuppression medications and combination regimens used during pregnancy in patients who had undergone liver transplantation for PSC. Data was not available in 5 patients.

	N=12
**Medications**	
Corticosteroid	9 (75%)
Ciclosporin	2 (17%)
Tacrolimus	10 (83%)
Azathioprine	8 (67%)
**Combination regimens**	
Tacrolimus monotherapy	2 (17%)
Tacrolimus and corticosteroid	2 (17%)
Tacrolimus and azathioprine	1 (8%)
Tacrolimus, corticosteroid and azathioprine	5 (42%)
Ciclosporin, corticosteroid and azathioprine	2 (17%)

Categorical variables are represented as number (%).
