# Supplementary material for: Maternal liver-related symptoms during pregnancy in primary sclerosing cholangitis
Source: JHEP Rep. 2023 Oct 31;6(1):100951. doi: 10.1016/j.jhepr.2023.100951 (PMC10711472; doi:10.1016/j.jhepr.2023.100951)
Supplement: Multimedia component 4 [file mmc4.pdf]

Research article

JHEP|Reports

Maternal liver-related symptoms during pregnancy in primary sclerosing cholangitis

Jeremy S. Nayagam,[1,2,*] Tobias J. Weismüller,[3,4] Piotr Milkiewicz,[5,6] Karolina M. Wronka,[5] Emil Bik,[5] Christoph Schramm,[7,8] Katja Fuessel,[7] Taotao Zhou,[3,8] Johannes Chang,[3,8] Martti Färkkilä,[8,9,10] Ylva Carlsson,[11,12] Anastasia Lundman,[13] Nora Cazzagon,[8,14] Giorgia Corrà,[8,14] Eirini Rigopoulou,[15] George N. Dalekos,[8,15] Aiva Lundberg Båve,[16] Annika Bergquist,[16] Karim Ben Belkacem,[17] Marco Marzioni,[18] Martina Mancinelli,[18] Xavier Verhelst,[8,19] Hanns-Ulrich Marschall,[8,13] Michael A. Heneghan,[2,8] Deepak Joshi [1,2], the International PSC Study Group (IPSCSG)

[1]Institute of Liver Studies, King's College Hospital, London, UK; [2]Department of Inflammation Biology, King's College London, London, UK; [3]Department of Internal Medicine I, University Hospital Bonn, Bonn, Germany; [4]Department of Internal Medicine - Gastroenterology and Oncology, Vivantes-Humboldt-Klinikum, Berlin, Germany; [5]Liver and Internal Medicine Unit, Medical University of Warsaw, Warsaw, Poland; [6]Translational Medicine Group, Pomeranian Medical University, Szczecin, Poland; [7]I.Department of Medicine and Martin Zeitz Center for Rare Diseases, University Medical Center Hamburg-Eppendorf, Hamburg, Germany; [8]European Reference Network for Hepatological Diseases (ERN RARE-LIVER), University Medical Center Hamburg-Eppendorf, Hamburg, Germany; [9]Clinic of Gastroenterology, Helsinki University Hospital, Helsinki, Finland; [10]Helsinki University, Helsinki, Finland; [11]Department of Obstetrics and Gynecology, Sahlgrenska University Hospital, Gothenburg, Sweden; [12]Sahlgrenska Academy, University of Gothenburg, Gothenburg, Sweden; [13]Department of Molecular and Clinical Medicine, University of Gothenburg, Gothenburg, Sweden; [14]Unit of Gastroenterology, Department of Surgery, Oncology and Gastroenterology, University Hospital of Padova, Padova, Italy; [15]Department of Medicine and Research Laboratory of Internal Medicine, National Expertise Center of Greece in Autoimmune Liver Diseases, General University Hospital of Larissa, Larissa, Greece; [16]Department of Medicine Huddinge, Unit of Gastroenterology and Rheumatology, Karolinska Institutet, Karolinska University Hospital, Stockholm, Sweden; [17]Reference Center for Inflammatory Biliary Diseases and Autoimmune Hepatitis, Saint-Antoine Hospital, Assistance Publique-Hôpitaux De Paris, Paris, France; [18]Clinic of Gastroenterology and Hepatology, Università Politecnica delle Marche, Ospedali Riuniti University Hospital, Ancona, Italy; [19]Department of Gastroenterology and Hepatology, Ghent University Hospital, Ghent, Belgium

JHEP Reports **2023.** https://doi.org/10.1016/j.jhepr.2023.100951

Background & Aims: Although worsening liver-related symptoms during pregnancy can occur in primary sclerosing cholangitis (PSC), there are insufficient data to effectively counsel patients on their pre-conception risk and no clear recommendations on monitoring and management during pregnancy. We aimed to describe maternal liver-related symptoms in pregnancy, both before and after PSC diagnosis, and explore factors associated with worsening symptoms and liver-related outcomes.

Methods: We conducted a multicentre retrospective observational study of females with PSC and known pregnancy with live birth, via the International PSC Study Group. We included 450 patients from 12 European centres. Data included clinical variables, liver-related symptoms (pruritus and/or cholangitis) during pregnancy, and liver biochemistry. A composite primary endpoint of transplant-free survival from time of PSC diagnosis was used.

Results: There were 266 pregnancies in 178 patients following PSC diagnosis. Worsening liver-related symptoms were reported in 66/228 (28.9%) pregnancies; they had a reduced transplant-free survival (p = 0.03), which retained significance on multivariate analysis (hazard ratio 3.02, 95% CI 1.24-7.35; p = 0.02).

Abnormal biochemistry and/or liver-related symptoms (pruritus and/or cholangitis) were noted during pregnancy before PSC diagnosis in 21/167 (12.6%) patients. They had a reduced transplant-free survival from pregnancy (p = 0.01), which did not retain significance in a multivariable model (hazard ratio 1.10, 95% CI 0.43-2.85; p = 0.84).

Conclusions: Liver-related symptoms are frequently encountered during pregnancies before the diagnosis of PSC, and pregnancy may expose the pre-clinical phase of PSC in some patients. Worsening liver-related symptoms were seen in a third of our cohort with known PSC during pregnancy; and this subgroup had a poorer prognosis, which may be related to more advanced liver disease at time of pregnancy and/or a more severe disease phenotype.

Impact and implications: Patients with PSC can develop worsening of their liver-related symptoms during pregnancy; however, risk factors for this and the long-term implications are not known. We identified that there is a significant risk of these symptoms in pregnancy, both before and after PSC has been diagnosed, particularly in patients with elevated alkaline phosphatase. Furthermore, our findings suggest that worsening symptoms during pregnancy may be associated with adverse long-term clinical outcomes of liver transplantation and death in patients with known PSC. This may

Keywords: Primary sclerosing cholangitis; Intrahepatic cholestasis of pregnancy; Cholangiopathy; Pruritus; Ursodeoxycholic acid; Bile acids.

Received 3 August 2023; received in revised form 4 October 2023; accepted 17 October 2023; available online XXX

* Corresponding author. Address: Institute of Liver Studies, King's College Hospital, London, UK. Tel: +44-203-299-3369.

E-mail address: j.nayagam@nhs.net (J.S. Nayagam).

ELSEVIER

EASL The Home of Hepatology

UNCORRECTED PROOF

be related to the presence of more advanced liver disease at time of pregnancy. This information can be used to counsel patients with PSC before conception and identify patients who need close follow-up after delivery.
© 2023 The Author(s). Published by Elsevier B.V. on behalf of European Association for the Study of the Liver (EASL). This is an open access article under the CC BY license (http://creativecommons.org/licenses/by/4.0/).

Introduction

Primary sclerosing cholangitis (PSC) is an immune-mediated chronic liver disease which is characterised by inflammation and fibrosis of the biliary tree, with development of multifocal biliary strictures and progressive hepatic fibrosis.[1] The natural history of PSC is highly variable and there are no currently licensed medications with a proven prognostic benefit in PSC. Furthermore, there is a significant life-time risk of progression to end-stage liver disease and development of hepatobiliary malignancies.[1]

PSC is commonly diagnosed at 25-40 years of age, and although there is male preponderance, a significant proportion of patients with PSC are females of child-bearing age during a period of peak fertility.[2] Maternal PSC is associated with an increased risk of pre-term birth and caesarean section; no demonstrable risk of stillbirth, neonatal death, or congenital abnormalities compared with the general population has been reported but this may reflect the paucity of data in the field.[3,4] Evaluating the effect of pregnancy on maternal liver disease is particularly pertinent in PSC, as pregnancy can have a variable effect on the course of immune-mediated diseases,[5] including inflammatory bowel disease (IBD)[6] which is common in PSC, and hormonal changes in pregnancy can contribute to the development of intrahepatic cholestasis of pregnancy (ICP).[7,8] Two studies reported maternal liver-related symptoms during pregnancy, with a combined 27 patients and 38 pregnancies, and identified: (i) a significant proportion of patients with new onset and/or worsening of symptoms (pruritus, abdominal pain) during pregnancy; and (ii) elevations in liver enzymes during pregnancy and post-partum.[9,10] This paucity of data on PSC-specific considerations during pregnancy hinders effective counselling before planned pregnancies, and the management and monitoring of patients during pregnancy and post-partum.[11,12]

We sought to address this through multicentre collaboration and description of maternal liver-related symptoms during pregnancy in patients with PSC. We hypothesised that patients with PSC are at increased risk of liver abnormalities during pregnancy, which can pre-date their diagnosis of PSC in the pre-clinical phase of PSC. Furthermore, we aimed to evaluate the effect of worsening symptoms during pregnancy on long-term maternal outcomes in patients with PSC.

Patients and methods

Study design

We conducted a multicentre retrospective observational study of female patients with PSC and known pregnancy. Members of the International PSC Study Group (IPSCSG) were invited to participate, and we received data from 12 centres in nine European countries (Belgium, Finland, France, Germany, Greece, Italy, Poland, Sweden, UK), which includes transplant and non-transplant tertiary liver centres. Female patients with a known live birth pregnancy at any time point in relation to their PSC diagnosis were included. Patients were excluded (Fig. S1) if they did not have a live birth pregnancy (n = 532) or if it was not known if they had a live birth pregnancy (n = 221).

The diagnosis of PSC and subtypes of PSC was confirmed in each centre in accordance with international guidelines.[12] All datasets were checked for plausibility and validity, including for duplicate entries based on identical patient identifiers, and outlying data was clarified with the individual centre. Ethical approval was obtained from King's College Hospital (19/WM/0145, IRAS 238575) in addition to local approval at participating centres. The study was supported by the European Association for the Study of the Liver Registry Grant (Liver Disease in Pregnancy).

Data collection

Baseline clinical variables were collected from the pre-existing IPSCSG database.[13] Each centre retrospectively examined the medical records of patients to update the IPSCSG database with clinical outcome data (liver transplantation [LT], death, hepatobiliary cancer diagnosis), details on pregnancy and liver biochemistry. Clinical outcome data was collected from pregnancy and diagnosis of PSC until death or last clinical follow-up alive. The study opened in March 2021 and follow-up data were collected until January 2022. Included patients were diagnosed with PSC between 1982 and 2021.

Symptoms during pregnancy, date of delivery, gestational age, mode of delivery, and need for urgent delivery as a result of liver disease were collected from review of case notes. In patients post-LT, additional information on pre-eclampsia, gestational diabetes and acute cellular rejection were collected. Details on ursodeoxycholic acid (UDCA) included use in pregnancies before PSC diagnosis, and whether it was continued, stopped, or started during pregnancies post-PSC diagnosis. Details on immunosuppression regimens were collected for pregnancies post-LT.

Liver biochemistry (total bilirubin, alkaline phosphatase [ALP], alanine aminotransferase [ALT], aspartate aminotransferase [AST], gamma-glutamyl transferase [GGT]) were collected where available at three time points: (i) peak value in the 6 months before conception; (ii) peak value during pregnancy; (iii) peak value in the 6 months after delivery. When performed, peak serum bile acids were also collected. The reference range of liver biochemistry for each laboratory value was obtained. For each Q5 individual the values are represented in reference to the upper limit of normal (ULN) and compared with their baseline pre-conception values. A threshold value of >25% rise from baseline pre-conception value was used, which was chosen as there is an intra-individual variability of ALP in PSC of 12-20% which is not associated with disease progression.[14]

Definitions

Liver abnormalities

In patients with pregnancies before their PSC diagnosis, the term 'liver abnormalities' was used for patients with: (i) a clinical diagnosis of ICP; (ii) abnormal liver biochemistry and/or liver-related symptoms during pregnancy without a diagnosis of ICP.

Worsening liver-related symptoms

Symptoms during pregnancies after PSC diagnosis were recorded as: (i) a clinical diagnosis of ICP; (ii) pruritus during pregnancy

UNCORRECTED PROOF

(worsened from baseline, stable/improved from baseline, newly developed or no pruritus); (iii) cholangitis during pregnancy (treated with antibiotics, treated with antibiotics and hospital admission, no cholangitis). Patients with known PSC were defined with '*worsening liver-related symptoms*' if they had a clinical diagnosis of ICP, new/worsening pruritus from pre-conception and/or an episode of cholangitis treated with antibiotics.

ICP

Diagnostic criteria for ICP differs between major societies,[15] therefore a clinical diagnosis by the treating clinicians was used.

Statistical analysis

Continuous variables are expressed as median (IQR), categorical variables are represented as number (percentage), or where data are missing number/number available (percentage). Subgroup differences were analysed using the X^2 or Fisher's exact tests for categorical parameters, and Mann-Whitney *U* test or Kruskal Wallis test for continuous variables. Unadjusted odds ratios (ORs) for developing worsening liver-related symptoms during pregnancy was calculated and recorded as OR with 95% 95% CIs and *p* values. Transplant-free survival (LT or death) was examined with Kaplan-Meier curves and the log-rank test. To determine predictors of LT or death during follow up, multivariate logistic regression analysis was performed to assess which features were of independent significance. Variables with a value of p <0.2 and those of particular interest were included. Results were recorded as hazard ratios (HRs) with 95% CIs and *p* values.

Statistical analysis of the data was performed using SPSS software (version 26, SPSS Inc, IBM, Armonk, NY, USA) and visualisation was performed using GraphPad Prism (version 9.5.1, San Diego, CA, USA). Two-sided *p* values <0.05 were considered statistically significant.

Results

Characteristics of study population

The patient cohort consisted of 450 females with PSC and their characteristics are outlined in Table 1. The median age at diagnosis was 33.8 years (IQR, 27.2-44.3 years), the majority had large duct PSC (n = 373, 82.9%) and concomitant IBD (n = 267, 59.3%), which was predominantly ulcerative colitis (n = 195, 43.4%). During follow up: 34 (7.6%) were diagnosed with hepatobiliary cancers (18 hepatocellular carcinoma, 23 cholangiocarcinoma, 5 gallbladder cancer), 104 (23.1%) underwent LT, and 46 (10.2%) died.

Pregnancy before PSC diagnosis

Maternal liver-related symptom data was available in 167 patients who were pregnant before their diagnosis of PSC, with a total of 299 pregnancies (Table S1). The median age at delivery was 28.7 years (IQR, 25.2-31.8 years), and subsequent PSC diagnosis 38.8 years (IQR, 32.4-48.1 years), with a median interval between delivery and PSC diagnosis of 5.8 years (IQR, 1.7-17.1 years). Most commonly there were 1 (n = 47, 28.1%) or 2 (n = 82, 49.1%) live births per patient before PSC diagnosis.

In 21 (12.6%) patients there was evidence of liver abnormalities during pregnancy before their PSC diagnosis: 10 (6.0%) ICP and 11 (6.6%) other (biochemistry and/or symptoms). UDCA was used in at least one pregnancy in seven (4.2%) patients. On univariate analysis there were no patient factors associated with development of liver abnormalities during a pregnancy before PSC diagnosis (Table 2); although they may have been younger at PSC diagnosis (34.6 [IQR, 32.1-39.3] *vs.* 40.7 [32.3-48.8] years; *p* = 0.06). There was a shorter interval between delivery and PSC diagnosis (1.4 [0.5-5.7] *vs.* 7.0 [2.0-18.0] years; *p* = 0.002) in patients with liver abnormalities.

Mean transplant-free survival from time of pregnancy was 33.9 years (95% CI 30.1-37.7, SE 1.9). There was a reduced 20-year transplant-free survival in those with liver abnormalities during pregnancy (*p* = 0.01; Fig. S2A). This however did not retain significance in a multivariate model (HR 1.10, 95% CI 0.43-2.85; *p* = 0.84), where age at diagnosis (HR 0.89, 95% CI 0.84-0.94; *p* <0.001) and development of large duct PSC (HR 5.16, 95% CI 1.13-23.51; *p* = 0.03) were associated with events of LT or death during follow up (Table 3).

Mean transplant-free survival from time of PSC diagnosis was 15.0 years (95% CI 13.2-16.8, SE 0.91). There was no difference in 20-year transplant-free survival between those with and without liver abnormalities during pregnancy (*p* = 0.21; Fig. S2B).

Pregnancy after PSC diagnosis

There were 266 pregnancies reported in 178 patients following PSC diagnosis (Table S2). The median age at PSC diagnosis was 26.8 years (IQR, 21.5-30.7 years), 146 (82.0%) had large duct PSC and 122 (66.5%) IBD. The median age at first delivery was 32.3 years (IQR, 28.7-35.7 years), at a median interval of 4.4 years (IQR, 2.2-8.9

Table 1. Clinical characteristics of study population with known live birth pregnancy and PSC (n = 450).

Number of patients	N = 450
Pregnancies	
Before PSC diagnosis	285 (63.3%)
After PSC diagnosis	178 (39.6%)
After liver transplant for PSC	14 (3.1%)
Age at diagnosis	
Median (IQR), years	33.8 (27.2-44.3)
≤20	41 (9.1%)
21-30	120 (26.7%)
31-40	136 (30.2%)
41-50	79 (17.6%)
51-60	48 (10.7%)
>60	20 (4.4%)
Unknown	6 (1.3%)
PSC subtype	
Large duct	373 (82.9%)
Small duct	29 (6.4%)
PSC/AIH overlap	46 (10.2%)
IgG4-SC	2 (0.4%)
IBD	267 (59.3%)
Ulcerative colitis	195 (43.3%)
Crohn's disease	55 (12.2%)
Indeterminate colitis	17 (3.8%)
No IBD	183 (40.7%)
Colectomy	43 (16.1%)
Malignancy	
Colorectal cancer	18 (4.0%)
Hepatobiliary cancer	34 (7.6%)
Cholangiocarcinoma	23 (5.1%)
Hepatocellular carcinoma	6 (1.3%)
Pancreatic cancer	0
Gallbladder cancer	5 (1.1%)
Outcome	
Liver transplantation	104 (23.1%)
Death	46 (10.2%)

Continuous variables are expressed as median (IQR), categorical variables are represented as number (%). AIH, autoimmune hepatitis; IBD, inflammatory bowel disease; IgG4-SC, immunoglobulin G4 sclerosing cholangitis; PSC, primary sclerosing cholangitis.

UNCORRECTED

Table 2. Univariate analysis of differences between patients with and without liver abnormalities detected during pregnancy before their PSC diagnosis. Q12

	Liver abnormalities (n = 21)	No liver abnormalities (n = 146)	*p* value
Age at PSC diagnosis, median (IQR), years	34.6 (32.1-39.3)	40.7 (32.3-48.8)	0.06
Age at delivery, median (IQR), years	30.7 (27.7-35.2)	28.6 (25.1-31.4)	0.09
Interval between delivery and PSC diagnosis, median (IQR), years	1.4 (0.5-5.7)	7.0 (2.0-18.0)	0.002
Large duct PSC	19 (90.5%)	113 (77.4%)	0.25
IBD	10 (47.6%)	75 (51.4%)	0.75

Continuous variables are expressed as median (IQR), categorical variables are represented as number (%). Subgroup differences were analysed using the X^2 or Fisher's exact tests for categorical parameters, and the Mann-Whitney *U* test or Kruskal Wallis test for continuous variables. IBD, inflammatory bowel disease; PSC, primary sclerosing cholangitis.

Table 3. Hazard ratio for events (LT or death) during follow up in patients with pregnancy before their PSC diagnosis.

	Univariate analysis			Multivariate analysis		
	HR	95% CI	*p value*	HR	95% CI	*p value*
Age at PSC diagnosis	0.89	0.85-0.93	<0.001	0.89	0.84-0.94	<0.001
Age at delivery	1.02	0.95-1.09	0.57			
Large duct PSC	6.23	1.49-26.21	0.01	5.16	1.13-23.51	0.03
IBD	3.22	1.55-6.66	0.002	1.46	0.64-3.30	0.36
Worsening liver-related symptoms during pregnancy	3.32	1.33-8.29	0.01	1.10	0.43-2.85	0.84

Multivariate logistic regression analysis was performed to assess which features were of independent significance. Variables with a *p* value of <0.2 and those of particular interest were included. Results were recorded as hazard ratio (HRs) with 95% CIs and *p* values. IBD, inflammatory bowel disease; PSC, primary sclerosing cholangitis.

years) from PSC diagnosis. UDCA was continued in 123 (46.2%) of pregnancies, stopped in 29 (10.9%), started in 11 (4.1%) and not used in 61 (22.9%); the remainder were unknown (42, 15.8%).

Peak liver biochemistry in the 6 months before conception, during pregnancy, and in the 6 months after delivery are listed in Table 4, both relative to the normal range and baseline pre-conception values. The most common abnormalities before conception and during pregnancy were elevations in AST and GGT; whereas after pregnancy it was GGT and ALP. A greater than 25% rise from baseline values was seen more commonly after delivery than during pregnancy in all liver biochemistry. We did not identify a difference in rise in liver biochemistry either during or after pregnancy in patients who continued taking UDCA during pregnancy (Table S3).

Maternal liver-related symptom data were available in 228 pregnancies. There was worsening of liver-related symptoms in 66 (28.9%) pregnancies: 33 (14.5%) clinical diagnosis of ICP, 23 (10.1%) worsening pruritus, 34 (14.9%) new pruritus, and four (1.8%) episode of cholangitis (Table S4). Serum bile acids were measured in a subgroup of 59 patients: 18/59 (30.5%) had elevated bile acids ≥40 µmol/L, including 8/59 (13.6%) with significant elevations at ≥100 µmol/L.

On univariate analysis continued UDCA use during pregnancy (OR 2.39 [1.10-5.22]; *p* = 0.03) and abnormal ALP before pregnancy (OR 4.40 [1.88-10.33]; *p* <0.001, Fig. 1) were associated with the development of liver-related symptoms during pregnancy (Table 5). There were no associations with age at delivery, subtype of PSC, duration of PSC or presence of IBD.

Mean transplant-free survival was 18.5 years (95% CI 17.9-19.2, SE 0.32). There was a reduced 20-year transplant-free survival in patients with pre-existing PSC who developed worsening liver-related symptoms during pregnancy (*p* = 0.03, Fig. 2). Age at diagnosis (HR 1.11, 95% CI 1.04-1.94; *p* = 0.003) and worsening liver-related symptoms during pregnancy (HR 3.02, 95% CI 1.24-7.35; *p* = 0.02) were patient factors associated with a reduced transplant-free survival and retained significance on multivariate analysis (Table 6). LT or death occurred at a median 8.1 years (IQR, 4.5-13.3 years) after pregnancy.

Pregnancy after LT for PSC

There were 17 pregnancies in 14 patients after LT for PSC, one patient had a diagnosis of recurrent PSC (rPSC) at the time of conception. The median age at delivery was 33.4 years (IQR, 27.5-36.7 years), time from transplant was 5.0 years (IQR, 2.5-10.8 years), and time from diagnosis of PSC 11.4 years (IQR, 7.6-14.9 years). Delivery was at a median 37.5 weeks gestation (range, 31-39 weeks). There were four spontaneous vaginal deliveries, two inductions, four elective caesarean sections, three emergency

Table 4. Peak values of liver biochemistry where available in the 6 months before conception, during pregnancy, and in the 6 months after delivery.

	Before conception		During pregnancy			After delivery		
	n	Normal range, n (%)	n	Normal range, n (%)	>25% rise from baseline	n	Normal range, n (%)	>25% rise from baseline
Bilirubin	115	95/115 (82.6%)	117	104/117 (88.9%)	18/96 (18.8%)	127	107/127 (84.3%)	30/96 (31.3%)
ALP	122	57/122 (46.7%)	119	57/119 (57.9%)	28/101 (27.7%)	131	36/131 (27.5%)	51/105 (48.6%)
ALT	75	35/75 (46.7%)	83	50/83 (60.2%)	13/67 (19.4%)	77	32/77 (41.6%)	29/62 (46.8%)
AST	86	30/86 (34.9%)	87	40/87 (46.0%)	17/72 (23.6%)	92	30/92 (32.6%)	30/73 (41.1%)
GGT	89	29/89 (32.6%)	84	40/84 (47.6%)	12/65 (18.5%)	96	26/96 (27.1%)	24/72 (33.3%)

The values are represented in reference to the upper limit of normal for the laboratory and compared with the individual's baseline pre-conception values. Categorical variables are represented as number (%), or where data are missing number/number available (%). ALP, alkaline phosphatase; ALT, alanine aminotransferase; AST, aspartate aminotransferase; GGT, gamma-glutamyl transferase.

UNCORRECTED PROOF

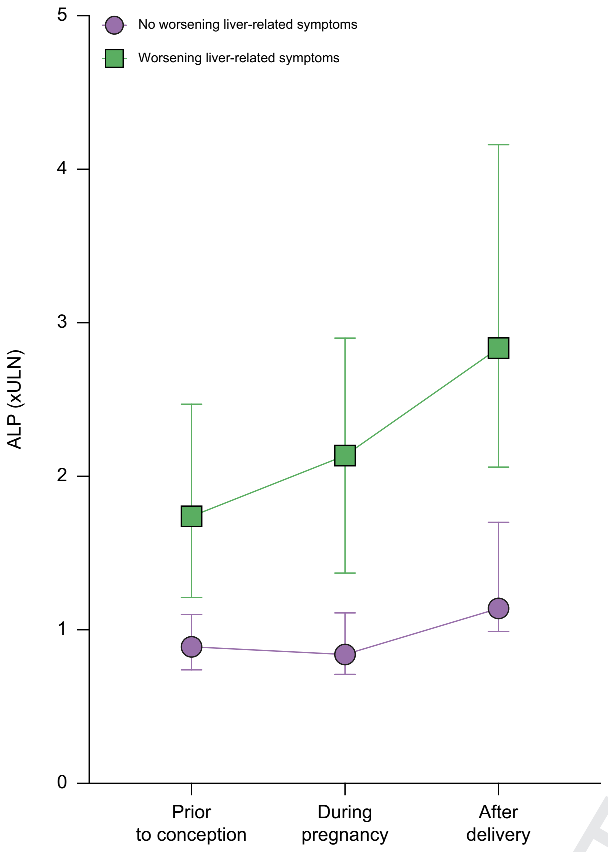

web 4C/FPO

Fig. 1. Serum ALP (in reference to the ULN for laboratory) expressed as median with 95% CI at three time points. (i) Peak value in the 6 months before conception; (ii) peak value during pregnancy; (iii) peak value in the 6 months after delivery. ALP, alkaline phosphatase; ULN, upper limit of normal.

caesarean sections and were unknown in four. Pre-eclampsia was diagnosed in two patients and no patients developed gestational diabetes.

The 12 pregnancies with complete data were maintained on calcineurin based immunosuppression during pregnancy (10 tacrolimus, two ciclosporin; Table S5); we were unable to ascertain the regimen in five patients. UDCA was continued during pregnancy in the three patients already taking it, and in the remainder it was not started. There were no episodes of acute cellular rejection during pregnancy.

In two of 17 (11.8%) pregnancies a clinical diagnosis of ICP was made, the remainder did not report liver-related symptoms during pregnancy. The patient with known rPSC before pregnancy was diagnosed with ICP and also had oral antibiotics for cholangitis; they subsequently required re-do LT 3 years post-partum.

Discussion

PSC is a rare disease with a paucity of data to inform women with PSC of the disease-specific risks associated with pregnancy, and to guide clinicians on their management during pregnancy.[11,12] In this retrospective European multicentre study we describe maternal liver-related symptoms and long-term outcomes in a large cohort of patients with PSC. We identified worsening of liver-related symptoms in a third of pregnant patients with PSC, which was associated with abnormal ALP before conception and UDCA use during pregnancy in our population. Furthermore, we demonstrated that patients who developed a worsening of their liver-related symptoms had a poorer transplant-free survival, and hypothesise that pregnancy-associated symptoms may have unmasked this subgroup with a more severe clinical phenotype and advanced liver disease. In addition, we reveal a novel finding that a significant proportion of patients with PSC had evidence of liver abnormalities during pregnancies before their PSC diagnosis.

We identified liver abnormalities in 12.6% of patients during pregnancy before their PSC diagnosis. This subgroup with liver abnormalities had a shorter interval to subsequent PSC diagnosis than those without, and were frequently diagnosed in the early post-partum period. Although no control group is available for comparison, a clinical diagnosis of ICP was made in 6% of our patients, which is greater than the 1% described in European populations.[16] Combining the ICP group with the heterogenous group of other liver abnormalities, we did not identify differences in baseline characteristics between patients who did and did not have liver abnormalities. ICP has been identified as a risk factor for future fibrosis/cirrhosis (HR 5.11, 95% CI 3.29-7.96), although this may be primarily influenced by chronic hepatitis C infection,[17] it is also likely to related to genetic factors that confer susceptibility to ICP and cirrhosis.[18,19] Although we demonstrated a poorer transplant-free survival from time of pregnancy in our cohort, there was no difference in transplant-free survival from time of PSC diagnosis (Fig. S2B) and this is likely to be related to a lead time bias and a shorter interval to PSC diagnosis. It has previously been reported that a small proportion of patients with ICP are subsequently diagnosed with PSC, with ICP a risk factor for future PSC diagnosis (HR 6.58, 95% CI 1.47-29.49).[17] It is debatable if all these patients truly had a diagnosis of ICP, particularly those diagnosed with PSC in the months after delivery. It is more plausible that it represents one or more of: (i) the natural history of their PSC; (ii) an unmasking of the preclinical phase of PSC[1] by hormonal changes in pregnancy;[7,8]

Table 5. Odds ratio developing worsening liver-related symptoms during pregnancy after a diagnosis of PSC (n = 228).

	Worsening liver-related symptoms (n = 66)	No worsening liver-related symptoms (n = 162)	*p* value	Odds ratio (95% CI)
Age at PSC diagnosis, median (IQR), years	25.8 (21.5-30.6)	25.8 (22.3-29.5)	0.68	1.009 (0.97-1.06)
Age at delivery, median (IQR), years	32.9 (29.0-36.0)	32.3 (29.2-35.6)	0.74	1.01 (0.95-1.07)
PSC duration at time of delivery, median (IQR), years	5.4 (3.3-9.4)	5.8 (2.9-10.2)	0.84	1.01 (0.95-1.06)
Large duct PSC	52/66 (78.8%)	132/162 (81.5%)	0.64	0.84 (0.42-1.72)
IBD	38/66 (57.6%)	109/162 (67.3%)	0.17	0.66 (0.37-1.19)
UDCA use	42/52 (80.8%)	79/124 (63.7%)	0.03	2.39 (1.10-5.22)
Abnormal bilirubin before pregnancy	8/39 (20.5%)	12/69 (17.4%)	0.69	1.23 (0.45-3.32)
Abnormal ALP before pregnancy	30/40 (75.0%)	30/74 (40.5%)	<0.001	4.40 (1.88-10.33)

Continuous variables are expressed as median (IQR), categorical variables are represented as number (%), or where data are missing number/number available (%). Unadjusted odds ratios (ORs) were calculated and recorded as OR with 95% 95% CIs and *p* values. ALP, alkaline phosphatase; IBD, inflammatory bowel disease; PSC, primary sclerosing cholangitis; UDCA, ursodeoxycholic acid.

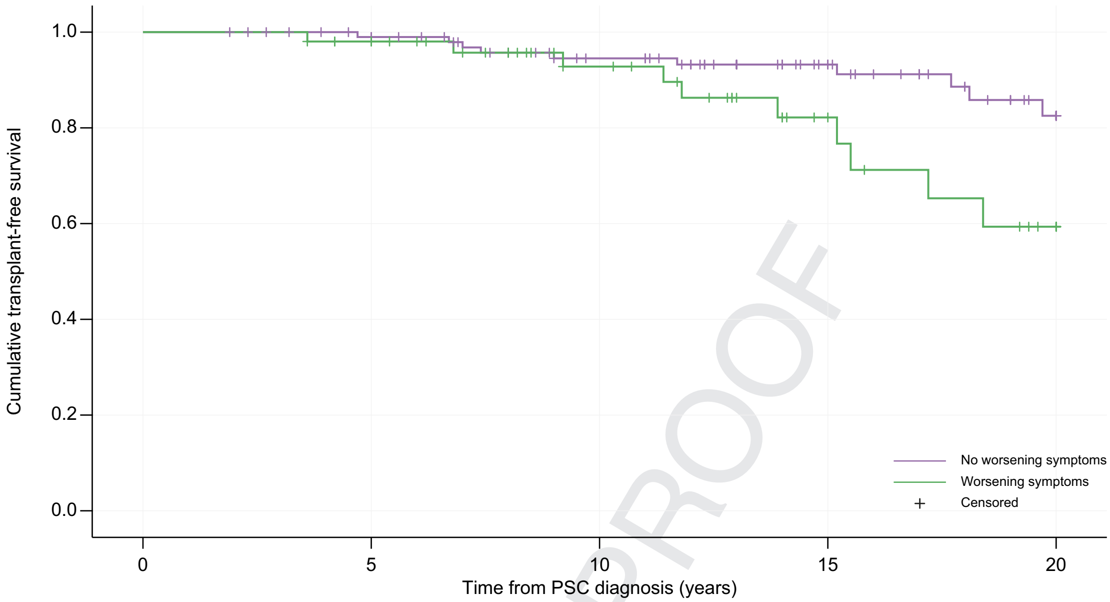

N° at risk					
Worsening symptoms	51	47	31	16	7
No worsening symptoms	104	97	77	49	25

web 4C/FPO

Fig. 2. Cumulative incidence of clinical events with Kaplan-Meier estimates for time to liver transplantation or death (transplant-free survival) from time of PSC diagnosis in those with pregnancy after PSC diagnosis. Twenty-year transplant-free survival in those with worsening liver-related symptoms in pregnancy (log-rank test, p = 0.03). PSC, primary sclerosing cholangitis.

Table 6. Hazard ratio for events (liver transplantation or death) during follow up in patients with pregnancy after their PSC diagnosis.

	Univariate analysis			Multivariate analysis		
	**HR**	**95% CI**	***p* value**	**HR**	**95% CI**	***p* value**
Age at diagnosis	1.10	1.03-1.18	0.005	1.11	1.04-1.94	0.003
Large duct PSC	0.44	0.10-1.88	0.27			
IBD	0.66	0.24-1.83	0.43			
Hepatobiliary cancer	0.66	0.15-2.86	0.58			
Worsening liver-related symptoms during pregnancy	2.61	1.08-6.30	0.03	3.02	1.24-7.35	0.02

Multivariate logistic regression analysis was performed to assess which features were of independent significance. Variables with a value of p <0.2 and those of particular interest were included. Results were recorded as hazard ratios (HRs) with 95% CIs and p values. IBD, inflammatory bowel disease; PSC, primary sclerosing cholangitis.

(iii) a genetic predisposition to abnormal bile acid homeostasis with variants in hepatobiliary transporters.[19] A more established cholangiopathy at time of pregnancy and/or a more severe phenotype of disease may be factors, however, these could not be explored in our study.

Worsening of liver-related symptoms were commonly reported during pregnancy in patients with PSC and affected 28.9% of our population, which is similar to previously reported smaller cohorts.[9,10] We explored pre-pregnancy factors associated with worsening symptoms and identified an elevated ALP pre-pregnancy and ongoing UDCA use during pregnancy. Although intra-individual variation in ALP does not associate with short-term progression in liver fibrosis,[14] an elevated ALP has been incorporated into prognostic models[20] and may represent the presence of more advanced liver disease and/or cholangiopathy, which could explain the association with worsening liver-related symptoms in our population. The association between UDCA use and worsening symptoms in pregnancy in our cohort is not clear; although UDCA is a well-recognised treatment for ICP which is associated with a reduction in symptoms[21] and may reduce spontaneous preterm birth in patients with elevated serum bile acids,[22] it does not clearly have a role in the standard treatment of PSC.[11,23] Although the short-term use of UDCA in patients with PSC and worsening symptoms during pregnancy has been suggested in guidelines,[11] our data do not add further light on the routine use of UDCA to prevent or reduce symptoms during pregnancy.

Liver biochemistry at the three time points (before conception, during pregnancy, and after delivery) were only available for a limited number of patients in this study, however there were sufficient numbers to demonstrate a clinical signal. Before conception the majority of our cohort had elevations in liver enzymes with a normal bilirubin. Significant elevations in liver biochemistry from pre-conception baseline, defined as >25% rise, were identified in 18-28% during pregnancy, and 31-49% after delivery. Physiological elevations in ALP are commonly seen in pregnancy related to placental secretion[24] which in part may explain the increases during pregnancy in our cohort. However, these elevations persisted after delivery, with a normal ALP in 46.7% before pregnancy compared to 27.5% at 6 months after delivery when ALP should be back to normal levels,[25] which may

UNCORRECTED PROOF

signify progressive liver disease. It was previously demonstrated in a smaller cohort of 25 pregnancies that there were more stable liver enzymes during pregnancy in patients continued on UDCA,[10] however we did not demonstrate a protective effect of UDCA on liver biochemistry abnormalities during pregnancy in our cohort. As the normal ranges for liver transaminases reduce in normal pregnancy, it is possible that the proportion of women with worsening liver biochemistry was underestimated in our study.

We demonstrated a prognostic impact of worsening liver-related symptoms during pregnancy, with a poorer transplant-free survival, which retained significance on multivariate analysis. Although the number of patients post LT in our cohort is small, the patient with known rPSC had a significant worsening of liver biochemistry during pregnancy, which progressed post-partum and they subsequently required re-transplantation. Unlike in patients pregnant before their PSC diagnosis, transplant-free survival should not be influenced by lead time bias, and this adds weight to the hypothesis that patients with a more advanced stage of liver disease and/or more severe phenotype of disease are at greater risk of developing worsening liver-related symptoms during pregnancy.

In addition to the challenges related to data collection and completeness of data, which is commonplace in large multi-centre retrospective studies, there are limitations specific to our study which need to be addressed. We only report a population with a diagnosis of PSC and live birth pregnancy and do not have a matched-control PSC population without pregnancy or other aetiologies of liver disease with pregnancy. Although we have been able to explore the effect of some factors associated on long-term outcomes, we do not have data on liver fibrosis assessment, presence of portal hypertension, and extent of cholangiopathy; all of which contribute to prognosis in PSC.[11,12] Liver-related symptoms were not systematically evaluated and may have been under-reported, particularly if pregnancies occurred at a different centre. As a retrospective analysis we were not able to assess for quality of life during pregnancy, and in particular symptoms common in both PSC and pregnancy such as fatigue and abdominal pain, which may have not been ascertained by their clinician. We only included pregnancies with live births and did not include pregnancies which resulted in foetal loss; this is an important subgroup to consider particularly with the association between severe ICP (bile acids ≥100 μmol/L) and foetal loss.[26] There is likely to have been significant variability in the clinical diagnosis of ICP in our population, related to the lack of consensus between the major societies for the diagnostic criteria of ICP,[15] and different thresholds for making a diagnosis of ICP in patients with established chronic liver disease. This is particularly challenging in PSC, where it is often not possible to distinguish between ICP and worsening cholestatic pruritus from underlying PSC, and we recommend caution in making a clinical diagnosis of ICP in PSC.

Pre-conception counselling is important in patients with chronic liver disease[27] and is recommended in PSC.[12] Prospective data with standardised monitoring during pregnancy is required to refine this information, however our study has provided important information to guide these discussions and can be used alongside recommendations in new international guidelines on liver disorders in pregnancy.[28] Pre-pregnancy biochemistry, such as elevations in ALP, can be used to help advise patients on their risk of developing new or worsening symptoms during pregnancy. Furthermore, patients who develop symptoms can be informed of the potential prognostic implications and need for closer monitoring in the medium and long-term. To truly personalise care and individualise risk before pregnancy, we envisage a multimodality assessment incorporating liver biochemistry, fibrosis assessment, quantification of cholangiopathy[29] and genetic testing of risk loci.[15,19] Recommendations on the frequency and benefit of blood tests during pregnancy for maternal liver-related complications cannot be derived from our data, however it is reasonable that they are checked in the second and third trimester, particularly with the association between elevated bile acids and poorer neonatal outcomes.[4]

In conclusion, we have demonstrated that a significant proportion of patients with PSC experience new or worsening liver-related symptoms during pregnancy, both before and after their diagnosis of PSC. Furthermore, we have identified that those who develop worsening liver-related symptoms during pregnancy may have a poorer long-term prognosis. Although the aetiopathogenesis of worsening liver-related symptoms during pregnancy in PSC is not clear and requires further evaluation, it may be related to the stage of liver disease at conception, with a 'stress test' from the hormonal changes during pregnancy which results in a clinical deterioration. We recommend pre-conception counselling for women with PSC, with close surveillance of those who develop worsening symptoms during pregnancy as they may have a more severe phenotype and/or advanced stage of disease.

UNCORRECTED PROOF

Abbreviations

AIH, autoimmune hepatitis; ALP, alkaline phosphatase; ALT, alanine aminotransferase; AST, aspartate aminotransferase; GGT, gamma-glutamyl transferase; HR, hazard ratio; IBD, inflammatory bowel disease; ICP, intrahepatic cholestasis of pregnancy; IgG4-SC, immunoglobulin G4 sclerosing cholangitis; LT, liver transplantation; OR, odds ratio; PSC, primary sclerosing cholangitis; rPSC, recurrent PSC; UDCA, ursodeoxycholic acid; ULN, upper limit of normal.

Financial support

Q7 ☐☐☐.

Conflicts of interest

The authors have no relevant disclosures.

Please refer to the accompanying ICMJE disclosure forms for further details.

Authors' contributions

Study concept and design: JSN, TJB, HUM, MAH, DJ. Acquisition of data: all authors

Analysis of data: JSN, DJ. Drafting of manuscript: JSN, MAH, DJ. Critical review of the manuscript: all authors.

Data availability statement

Data from the current study are available from the corresponding author on reasonable request.

Acknowledgements

International Primary Sclerosing Cholangitis Study Group; European Reference Network - Rare Liver; European Association for the Study of the Liver Registry Grant (Liver Disease in Pregnancy).

Supplementary data

Supplementary data to this article can be found online at https://doi.org/10.1016/j.jhepr.2023.100951.

References

Author names in bold designate shared co-first authorship

Q8 Q9 Q11 Q10

[1] Karlsen TH, Folseraas T, Thorburn D, Vesterhus M. Primary sclerosing cholangitis-a comprehensive review. J Hepatol 2017;67:1298–1323.
[2] Zakharia K, Tabibian A, Lindor KD, Tabibian JH. Complications, symptoms, quality of life and pregnancy in cholestatic liver disease. Liver Int 2018;38:399–411.
[3] Ludvigsson JF, Bergquist A, Ajne G, Kane S, Ekbom A, Stephansson O. A population-based cohort study of pregnancy outcomes among women with primary sclerosing cholangitis. Clin Gastroenterol Hepatol 2014;12:95–100.
[4] Cauldwell M, Mackie FL, Steer PJ, Henehghan MA, Baalman JH, Brennand J, et al. Pregnancy outcomes in women with primary biliary cholangitis and primary sclerosing cholangitis: a retrospective cohort study. BJOG 2020;127:876–884.
[5] Piccinni MP, Lombardelli L, Logiodice F, Kullolli O, Parronchi P, Romagnani S, et al. How pregnancy can affect autoimmune diseases progression? Clin Mol Allergy 2016;14:1–9.
[6] Selinger CP, Nelson-Piercy C, Fraser A, Hall V, Limdi J, Smith L, et al. IBD in pregnancy: recent advances, practical management. Frontline Gastroenterol 2021;12:214–224.
[7] Abu-Hayyeh S, Papacleovoulou G, Lövgren-Sandblom A, Tahir M, Oduwole O, Jamaludin NA, et al. Intrahepatic cholestasis of pregnancy levels of sulfated progesterone metabolites inhibit farnesoid X receptor resulting in a cholestatic phenotype. Hepatology 2013;57:716–726.
[8] Song X, Vasilenko A, Chen Y, Valanejad L, Verma R, Yan B, et al. Transcriptional dynamics of bile salt export pump during pregnancy: mechanisms and implications in intrahepatic cholestasis of pregnancy. Hepatology 2014;60:1993–2007.
[9] Janczewska I, Olsson R, Hultcrantz R, Broomé U. Pregnancy in patients with primary sclerosing cholangitis. Liver 1996;16:326–330.
[10] Wellge BE, Sterneck M, Teufel A, Rust C, Franke A, Schreiber S, et al. Pregnancy in primary sclerosing cholangitis. Gut 2011;60:1117–1121.
[11] Chapman MH, Thorburn D, Hirschfield GM, Webster GGJ, Rushbrook SM, Alexander G, et al. British Society of Gastroenterology and UK-PSC guidelines for the diagnosis and management of primary sclerosing cholangitis. Gut 2019;68:1356–1378.
[12] European Association for the Study of the Liver. EASL clinical practice guidelines on sclerosing cholangitis. J Hepatol 2022;77:761–806.
[13] Weismuller TJ, Trivedi PJ, Bergquist A, Imam M, Lenzen H, Ponsioen CY, et al. Patient age, sex, and inflammatory bowel disease phenotype associate with course of primary sclerosing cholangitis. Gastroenterology 2017;152:1975–1984.e8.
[14] Trivedi PJ, Muir AJ, Levy C, Bowlus CL, Manns MP, Lu X, et al. Inter-and intra-individual variation, and limited prognostic utility, of serum alkaline phosphatase in a trial of patients with primary sclerosing cholangitis. Clin Gastroenterol Hepatol 2021;19:1248–1257.
[15] Bicocca MJ, Sperling JD, Chauhan SP. Intrahepatic cholestasis of pregnancy: review of six national and regional guidelines. Eur J Obstet Gynecol Reprod Biol 2018;231:180–187.
[16] Geenes V, Williamson C. Intrahepatic cholestasis of pregnancy. World J Gastrenterol 2009;15:2049.
[17] Marschall HU, Wikström Shemer E, Ludvigsson JF, Stephansson O. Intrahepatic cholestasis of pregnancy and associated hepatobiliary disease: a population-based cohort study. Hepatology 2013;58:1385–1391.
[18] Dixon PH, Sambrotta M, Chambers J, Taylor-Harris P, Syngelaki A, Nicolaides K, et al. An expanded role for heterozygous mutations of ABCB4, ABCB11, ATP8B1, ABCC2 and TJP2 in intrahepatic cholestasis of pregnancy. Sci Rep 2017;7:11823.
[19] Nayagam JS, Williamson C, Joshi D, Thompson RJ. Liver disease in adults with variants in the cholestasis-related genes ABCB11, ABCB4 and ATP8B1. Aliment Pharmacol Ther 2020;52:1628–1639.
[20] Goode EC, Clark AB, Mells GF, Srivastava B, Spiess K, Gelson WTH, et al. Factors associated with outcomes of patients with primary sclerosing cholangitis and development and validation of a risk scoring system. Hepatology 2019;69:2120–2135.
[21] Bacq Y, Sentilhes L, Reyes HB, Glantz A, Kondrackiene J, Binder T, et al. Efficacy of ursodeoxycholic acid in treating intrahepatic cholestasis of pregnancy: a meta-analysis. Gastroenterology 2012;143:1492–1501.
[22] Ovadia C, Sajous J, Seed PT, Patel K, Williamson NJ, Attilakos G, et al. Ursodeoxycholic acid in intrahepatic cholestasis of pregnancy: a systematic review and individual participant data meta-analysis 2021. Lancet Gastroenterol Hepatol 2021;6:547–558.
[23] Triantos CK, Koukias NM, Nikolopoulou VN, Burroughs AK. Meta-analysis: ursodeoxycholic acid for primary sclerosing cholangitis. Aliment Pharmacol Ther 2011;34:901–910.
[24] Joshi D, James A, Quaglia A, et al. Liver disease in pregnancy. Lancet 2010;375:594–605.
[25] Larsson A, Palm M, Hansson LO, Axelsson O. Reference values for clinical chemistry tests during normal pregnancy. BJOG 2008;115:874–881.
[26] Ovadia C, Seed PT, Sklavounos A, Geenes V, Di Ilio D, Chambers J, et al. Association of adverse perinatal outcomes of intrahepatic cholestasis of pregnancy with biochemical markers: results of aggregate and individual patient data meta-analyses. Lancet 2019;393:899–909.
[27] Rahim MN, Pirani T, Williamson C, Heneghan MA. Management of pregnancy in women with cirrhosis. United Eur Gastroenterol J 2021;9:110–119.
[28] European Association for the Study of the Liver. EASL Clinical Practice Guidelines on the management of liver diseases in pregnancy. J Hepatol 2023;79:768–828.
[29] Grigoriadis A, Ringe KI, Bengtsson J, Baubeta E, Forsman C, Korsavidou-Hult K, et al. Development of a prognostic MRCP-score (DiStrict) for individuals with large-duct primary sclerosing cholangitis. JHEP Rep 2022;4:100595.

UNCORRECTED PROOF

Journal of Hepatology, Volume ■

Supplemental information

Maternal liver-related symptoms during pregnancy in primary sclerosing cholangitis

Jeremy S. Nayagam, Tobias J. Weismüller, Piotr Milkiewicz, Karolina M. Wronka, Emil Bik, Christoph Schramm, Katja Fuessel, Taotao Zhou, Johannes Chang, Martti Färkkilä, Ylva Carlsson, Anastasia Lundman, Nora Cazzagon, Giorgia Corrà, Eirini Rigopoulou, George N. Dalekos, Aiva Lundberg Båve, Annika Bergquist, Karim Ben Belkacem, Marco Marzioni, Martina Mancinelli, Xavier Verhelst, Hanns-Ulrich Marschall, Michael A. Heneghan, Deepak Joshi, and the International PSC Study Group (IPSCSG

Maternal liver-related symptoms during pregnancy in primary sclerosing cholangitis

Jeremy S Nayagam, Tobias J Weismüller, Piotr Milkiewicz, Karolina M Wronka, Emil Bik, Christoph Schramm, Katja Fuessel, Taotao Zhou, Johannes Chang, Martti Färkkilä, Ylva Carlsson, Anastasia Lundman, Nora Cazzagon, Giorgia Corrà, Eirini Rigopoulou, George N Dalekos, Aiva Lundberg Båve, Annika Bergquist, Karim Ben Belkacem, Marco Marzioni, Martina Mancinelli, Xavier Verhelst, Hanns-Ulrich Marschall, Michael A Heneghan, Deepak Joshi, the International PSC Study Group (IPSCSG)

Table of contents

Fig. S1……………………………………………………………………………………2

Fig. S2……………………………………………………………………………………3

Table S1…………………………………………………………………………………5

Table S2…………………………………………………………………………………6

Table S3…………………………………………………………………………………7

Table S4…………………………………………………………………………………9

Table S5…………………………………………………………………………………10

Fig. S1. Study design and subject disposition.

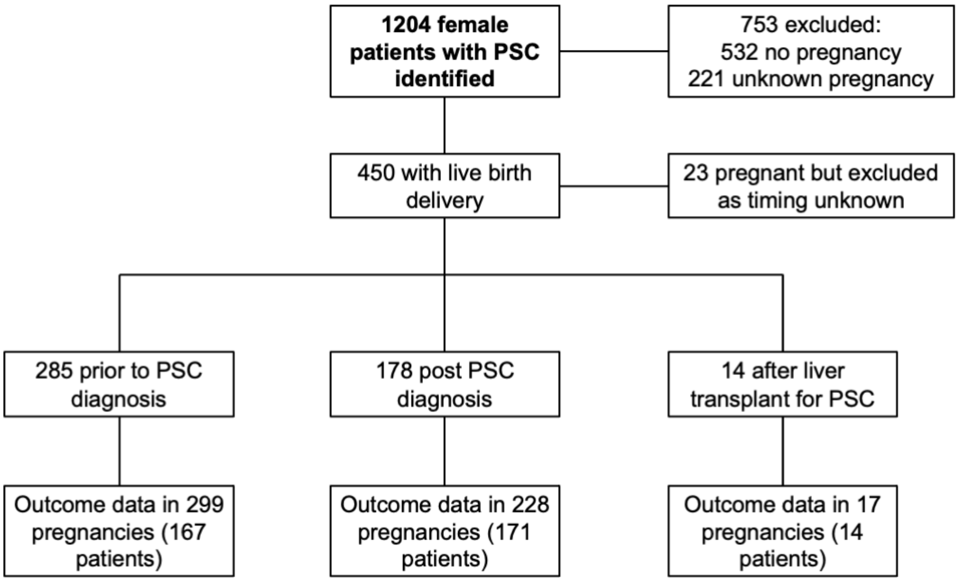

Fig. S2A. Cumulative incidence of clinical events with Kaplan-Meier estimates for time to liver transplantation or death (transplant-free survival) from time of pregnancy prior to PSC diagnosis. 20 year transplant-free survival in those with liver abnormalities in pregnancy (log-rank, P=0.01).

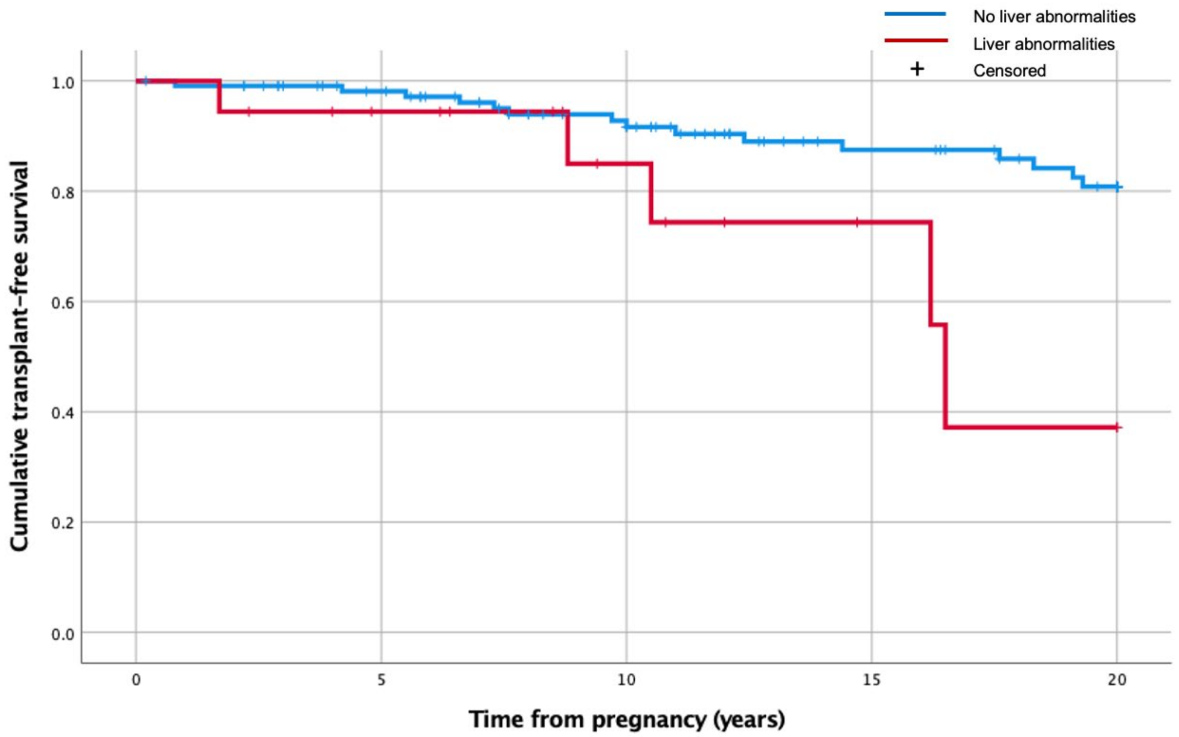

At risk					
Liver abnormalities	*18*	14	8	4	2
No liver abnormalities	*113*	100	81	58	47

Fig. S2B. Cumulative incidence of clinical events with Kaplan-Meier estimates for time to liver transplantation or death (transplant-free survival) from time of PSC diagnosis in those with pregnancy prior to diagnosis. 20 year transplant-free survival in those with liver abnormalities in pregnancy (log-rank, P=0.21).

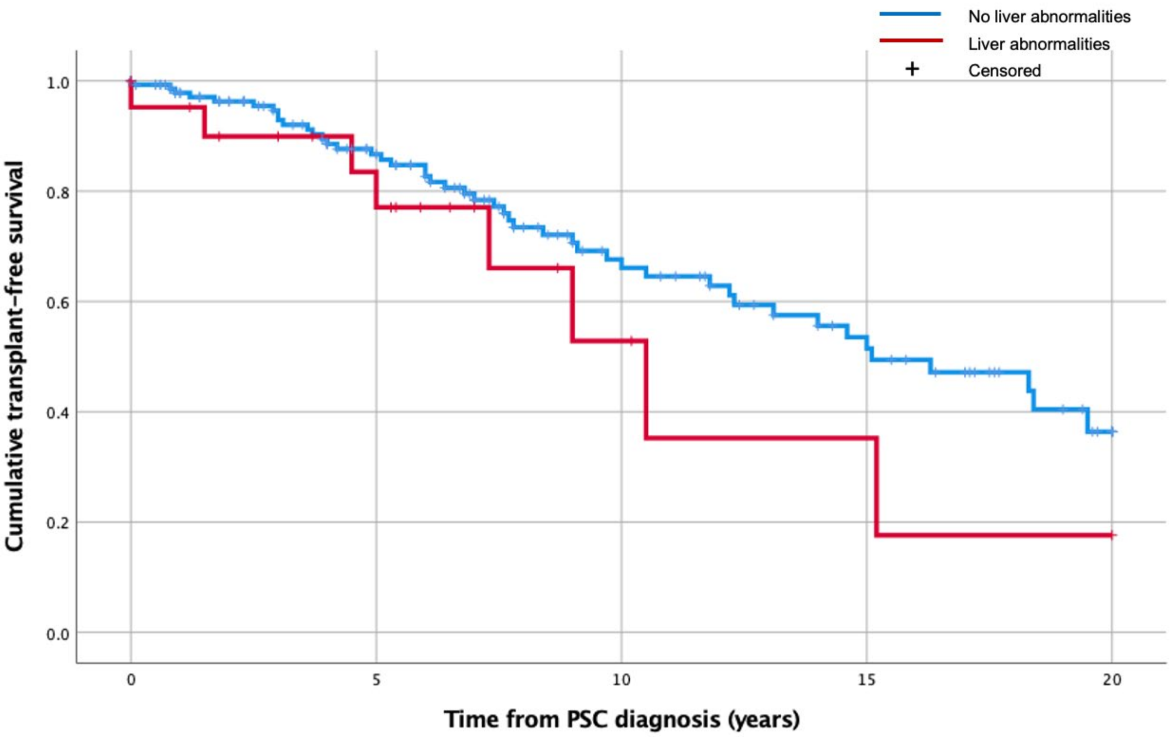

At risk					
Liver abnormalities	21	13	4	2	1
No liver abnormalities	146	90	44	26	7

Table S1. Clinical characteristics of subgroup of patients who were pregnant prior to their diagnosis of PSC (n=167).

	N=167
**Age at delivery, median (IQR), years**	28.7 (25.2-31.8),
**Age at PSC diagnosis, median (IQR), years**	38.8 (32.4-48.1)
**Interval between delivery and PSC diagnosis, median (IQR), years**	5.8 (1.7-17.1)
**Large duct**	132 (79.0%)
**Small duct**	14 (8.4%)
**PSC/AIH overlap**	19 (11.4%)
**IgG4-SC**	2 (1.2%)
**IBD**	85 (50.9%)

Abbreviations: PSC, primary sclerosing cholangitis; AIH, autoimmune hepatitis; IgG4-SC, immunoglobulin G4 sclerosing cholangitis; IBD, inflammatory bowel disease.

Continuous variables are expressed as median (IQR), categorical variables are represented as number (%).

Table S2. Clinical characteristics of subgroup of patients who were pregnant after their diagnosis of PSC (n=178).

	N=178
**Age at PSC diagnosis, median (IQR), years**	26.8 (21.5-30.7)
**Age at delivery, median (IQR), years**	32.3 (28.7-35.7)
**PSC duration at time of delivery, median (IQR), years**	4.4 (2.2-8.9)
**Large duct**	146 (82.0%)
**Small duct**	8 (4.5%)
**PSC/AIH overlap**	24 (13.5%)
**IBD**	122 (66.5%)

Abbreviations: PSC, primary sclerosing cholangitis; AIH, autoimmune hepatitis; IBD, inflammatory bowel disease.
Continuous variables are expressed as median (IQR), categorical variables are represented as number (%).

Table S3. Univariate analysis of continued UDCA use during pregnancy and change in liver blood tests from baseline values.

	UDCA not used previously or stopped	UDCA continued	P
**Bilirubin**			
Normal prior to conception	33/38 (86.8%)	53/65 (81.5%)	0.48
>25% rise from baseline during pregnancy	4/34 (11.8%)	10/51 (19.6%)	0.34
>25% rise post from baseline after delivery	8/32 (25.0%)	17/53 (32.1%)	0.49
**ALP**			
Normal prior to conception	19/42 (45.2%)	33/68 (48.5%)	0.74
>25% rise from baseline during pregnancy	7/36 (19.4%)	16/55 (29.1%)	0.30
>25% rise post from baseline after delivery	17/36 (47.2%)	27/58 (46.6%)	0.95
**ALT**			
Normal prior to conception	9/22 (40.9%)	24/46 (52.2%)	0.39
>25% rise from baseline during pregnancy	2/20 (10.0%)	7/41 (17.1%)	0.70
>25% rise post from baseline after delivery	8/29 (27.6%)	12/34 (35.3%)	0.51
**AST**			
Normal prior to conception	14/32 (43.8%)	12/42 (28.6%)	0.18
>25% rise from baseline during pregnancy	5/30 (16.7%)	6/32 (18.8%)	0.83

>25% rise post from baseline after delivery	10/18 (55.6%)	16/39 (41.0%)	0.31
**GGT**			
Normal prior to conception	9/27 (33.3%)	16/51 (31.4%)	0.86
>25% rise from baseline during pregnancy	**7/23 (30.4%)**	**3/35 (8.6%)**	**0.04**
>25% rise post from baseline after delivery	9/24 (37.5%)	8/39 (20.5%)	0.14

Abbreviations; UDCA, ursodeoxycholic acid; ALP, alkaline phosphatase; ALT, alanine aminotransferase; AST, aspartate aminotransferase; GGT, gamma-glutamyl transferase.

Categorical variables are represented as number (%), or where data is missing number/number available (%). Subgroup differences were analysed by Chi-squared or Fisher's exact tests.

Table S4. Reported symptoms during pregnancy after PSC diagnosis (n=228).

	N=228
**Clinical diagnosis of intrahepatic cholestasis of pregnancy**	33 (14.5%)
**Pruritus**	
Stable or improved from baseline	33 (14.5%)
Worsened from baseline	23 (10.1%)
Newly developed	34 (14.9%)
**Cholangitis**	
Treated with antibiotics as outpatient	3 (1.3%)
Treated with antibiotics as inpatient	1 (0.4%)
**Any worsening liver-related symptom during pregnancy**	66 (28.9%)

Categorical variables are represented as number (%).

Table S5. Immunosuppression medications and combination regimens used during pregnancy in patients who had undergone liver transplantation for PSC. Data was not available in 5 patients.

	N=12
**Medications**	
Corticosteroid	9 (75%)
Ciclosporin	2 (17%)
Tacrolimus	10 (83%)
Azathioprine	8 (67%)
**Combination regimens**	
Tacrolimus monotherapy	2 (17%)
Tacrolimus and corticosteroid	2 (17%)
Tacrolimus and azathioprine	1 (8%)
Tacrolimus, corticosteroid and azathioprine	5 (42%)
Ciclosporin, corticosteroid and azathioprine	2 (17%)

Categorical variables are represented as number (%).
